# Characteristics and Propagation of Airgun Pulses in Shallow Water with Implications for Effects on Small Marine Mammals

**DOI:** 10.1371/journal.pone.0133436

**Published:** 2015-07-27

**Authors:** Line Hermannsen, Jakob Tougaard, Kristian Beedholm, Jacob Nabe-Nielsen, Peter Teglberg Madsen

**Affiliations:** 1 Section for Marine Mammal Research, Department of Bioscience, Aarhus University, Roskilde, Denmark; 2 Zoophysiology, Department of Bioscience, Aarhus University, Aarhus, Denmark; 3 Murdoch University Cetacean Research Unit, School of Veterinary and Life Sciences, Murdoch University, Perth, Australia; University of St Andrews, UNITED KINGDOM

## Abstract

Airguns used in seismic surveys are among the most prevalent and powerful anthropogenic noise sources in marine habitats. They are designed to produce most energy below 100 Hz, but the pulses have also been reported to contain medium-to-high frequency components with the potential to affect small marine mammals, which have their best hearing sensitivity at higher frequencies. In shallow water environments, inhabited by many of such species, the impact of airgun noise may be particularly challenging to assess due to complex propagation conditions. To alleviate the current lack of knowledge on the characteristics and propagation of airgun pulses in shallow water with implications for effects on small marine mammals, we recorded pulses from a single airgun with three operating volumes (10 in^3^, 25 in^3^ and 40 in^3^) at six ranges (6, 120, 200, 400, 800 and 1300 m) in a uniform shallow water habitat using two calibrated Reson 4014 hydrophones and four DSG-Ocean acoustic data recorders. We show that airgun pulses in this shallow habitat propagated out to 1300 meters in a way that can be approximated by a 18log(r) geometric transmission loss model, but with a high pass filter effect from the shallow water depth. Source levels were back-calculated to 192 dB re µPa^2^s (sound exposure level) and 200 dB re 1 µPa dB L_eq-fast_ (rms over 125 ms duration), and the pulses contained substantial energy up to 10 kHz, even at the furthest recording station at 1300 meters. We conclude that the risk of causing hearing damage when using single airguns in shallow waters is small for both pinnipeds and porpoises. However, there is substantial potential for significant behavioral responses out to several km from the airgun, well beyond the commonly used shut-down zone of 500 meters.

## Introduction

Anthropogenic activity and encroachment in marine areas are rapidly increasing. This causes an elevation in underwater noise levels with potential negative impacts on marine species, such as displacement from favorable areas, masking of important signals and in the worst cases auditory impairment or physical injury [1,2,3]. Anthropogenic underwater noise stems from a range of activities of which shipping, offshore construction and geophysical exploration are the most widespread and energetic sources [1,4,5]. Geophysical exploration, including seismic surveys, are used for characterizing geological layers in the seabed via emission of low frequency sound pulses from airguns [6] that are among the most powerful anthropogenic noise sources in the marine environment [1]. Because of their high intensities at low frequencies, airgun pulses have been detected above ambient noise levels out to ranges of several thousand kilometers in deep water [7]. Airguns are normally used in a horizontal array configuration designed to direct a low frequency sound pulse at the seabed by means of beam forming. However, the array also radiates sound energy in other directions as the individual airguns are omnidirectional sound sources. Furthermore, even though airgun arrays are designed to produce a low frequency pulse centered around 50 Hz [4], the inherently broadband nature of a transient sound source in concert with a very high source level give rise to a very broad frequency spectrum [4,6].

Because most energy is found at low frequencies in airgun pulses, studies of seismic noise impacts on marine mammals have focused primarily on the effects on large baleen whales and sperm whales [8,9,10,11], whose (presumed) better low frequency hearing [12,13] most likely overlaps more with the peak frequency of the airguns. However, reports of substantial energy at medium-to-high frequencies [14,15,16,17] have raised concern that seismic noise may also have the potential to adversely affect smaller marine mammals with more sensitive hearing at higher frequencies [13]. Substantiating this concern, seismic surveys have been associated with increased stress hormone levels in belugas and bottlenose dolphins [18], ceased feeding and bradycardia in harbor seals [19], reduced feeding activity in harbor porpoises [20] and strandings in beaked whales [21,22]. Additionally, observations from seismic vessels by Stone and Tasker [23] and Weir [24] suggest that small toothed whales may even show stronger avoidance responses to airgun pulses than do larger cetaceans, despite their generally much poorer low frequency hearing [13].

In shallow coastal waters in particular, airgun noise may contain proportionally more energy at medium-to-high frequencies, where smaller toothed whales hear well. This is caused by the poor propagation of low frequencies in shallow water environments that act as high pass filters [1,4]. Multipath propagation will also cause patterns of constructive and destructive interference of sounds bouncing back and forth between the surface and bottom, which in conjunction with ray bending effects from a variable sound velocity profile and sound propagation in the sea bed result in non-trivial changes to the spectral and temporal characteristics of noise with increasing distance to the source [25,26]. All these effects make it difficult to estimate the characteristics and propagation of such low frequency noise sources in shallow water and hence assess the impact ranges for marine mammals. Noise exposure levels should therefore be based on, or at least verified against, actual measurements and not based solely on source levels and a few environmental properties [27]. Only few studies have dealt with propagation of airgun noise in shallow water habitats. Greene and Richardson [28] recorded airguns in water depths of 9˗130 m with recordings indicating elevated noise levels above 500 Hz (their maximum analysis frequency) from the pulses out to at least 11 km. A decade later, a higher sampling rate enabled Goold and Fish [14] to show noise components up to 22 kHz at a range of 2 km from a 2120 in^3^ seismic array in water depths of 50–100 m. Guerra et al. [29] recorded sound from a 3147 in^3^ airgun survey in 20–50 m water depth and found elevated noise levels even at a range of 128 km from the survey, but the authors focused on reverberations and the pulses were only analyzed up to 450 Hz.

In shallow waters, single airguns or small airgun arrays are used to characterize the topmost layers of the seabed (sub-bottom profiling) in connection with offshore construction, cable laying and oil and gas drilling [4,30]. Yet, to our knowledge only the study by Greene and Richardson [28] has investigated the characteristics and propagation of noise from a single airgun in shallow water, however with focus on frequencies below 500 Hz. To alleviate the lack of data pertinent to small odontocetes, we report here the source characteristics and propagation of broadband pulses (10 Hz up to 120 kHz) from a small airgun operated with three different volumes and at different firing pressure in a shallow water habitat. These data are discussed in relation to the potential effects on two key species from shallow water: harbor porpoise (*Phocoena phocoena*) and harbor seal (*Phoca vitulina*). Both species are often found in shallow waters with high seismic survey intensity, such as the North Sea [31].

## Methods

### Recordings

Recordings of airgun noise were obtained at sea state 0 in Aarhus Bay (along a line from 56°07.45N, 10°18.24E to 56°06.74N, 10°19.11E) over a sandy bottom with a uniform water depth of 15 m (± 0.2 m). An airgun with three volume options (sleeve gun-I 40 in^3^ with chamber inserts to reduce volume to 10 and 25 in^3^; HGS, Halliburton Geophysical Services) was fired in mid-water (7.5 m) from an anchored research vessel (R/V Tyra). The airgun was first fired four to five times at air pressures of 120–122 bar for the 10 in^3^ and 40 in^3^ volume and 114–117 bar for the 25 in^3^ volume, and then every 10 seconds with decreasing air pressure in the airgun ending with four to five pulses at low pressure (53–56 bar). The airgun pulses were recorded at mid-water simultaneously at six ranges: 6, 120, 200, 400, 800 and 1300 m. At the two closest stations (6 m and 120 m) recordings were made with Reson TC4034 hydrophones (Reson, Slangerup, Denmark; sensitivity -218 dB re 1V/μPa with a flat frequency response (±3 dB) from 10 to 250 kHz). Hydrophones were fitted with 3 kg lead weights to reduce movement. Signals were amplified 10 dB (at 6 m) or 20 dB (at 120 m) with a low-noise amplifier (A1001, ETEC, Frederiksværk, Denmark, 1-pole, 10 Hz high pass filter or EC6081/VP2000 voltage preamplifier, Reson, Slangerup, Denmark, 1-pole, 1 Hz high pass filter and 4-pole, 250 kHz low pass filter). Recordings were made with 16 bit analog-to-digital converters sampling at 500 kHz (National Instruments USB-6251 and USB-6356) and laptops running custom made software (LabVIEW, National Instruments, 2011, courtesy of Alain Moriat). At the remaining stations (200, 400, 800 and 1300 m from the airgun) recordings were obtained at mid-water depth with DSG-Ocean acoustic data recorders (Loggerhead Instruments, Sarasota, Florida; sensitivity -210 dB re 1V/μPa, flat frequency response up to 35 kHz, 1-pole high-pass filter at 10 Hz, 3-pole low pass filter at 35 kHz, 10 dB gain) sampling at 40 kHz with 16 bit resolution. None of the recorded airgun pulses were clipped. The range between the airgun and each recording station was calculated from GPS-derived positions on all deployment locations.

No permission to conduct the fieldwork was obtained, as this was not required under Danish law. Nevertheless, to reduce the risk of adverse effects to marine mammals, in particular harbor porpoises, an Airmar pinger (10 kHz; 132 dB re 1 μPa pp; 4 s interval) was deployed 30 minutes prior to airgun trials.

### Analysis

**Fig 1:**
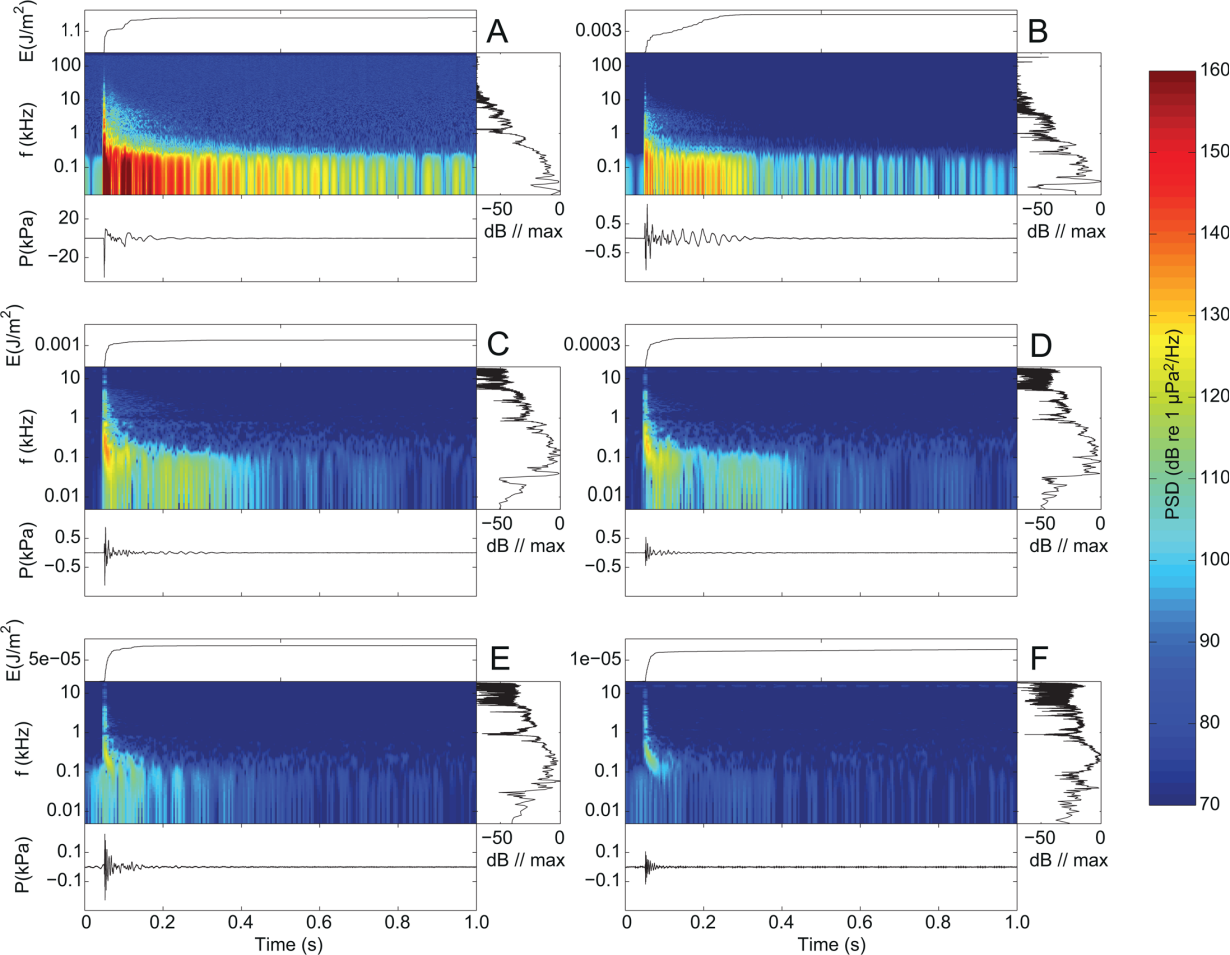
Changes to the airgun signal with increasing range. Shown are representative recordings of the 40 in^3^ airgun with approx. 120 bar output pressure obtained at different ranges from the airgun. A: 6 m, B: 120 m, C: 200 m, D: 400 m, E: 800 m, and F: 1300 m. See legend to Fig 1 for explanation of subplots. Note that the y-axis on the center panel extends above 100 kHz for plot A and B compared to 10 kHz for the remaining plots, because of differences in sampling rates for the six stations. Note also the different y-axis scales in the time plots (top and bottom panels).

Individual airgun pulses were analyzed in 1 second windows starting 50 ms before pulse onset. All recordings were high pass filtered with a fourth order Butterworth filter at 5 Hz. Spectrograms were computed after low-pass filtering with a fourth order Butterworth filter at either 200 kHz (ranges 6 m and 120 m) or 18 kHz (ranges 200 m and above). A very small, recurrent electrical glitch (visible around 15 kHz in Fig 1) was coherently removed from the signals. Removal of this artefact changed the end results by less than 1 dB. All analyses were done in MATLAB (MATHWORKS 7.5.0).

A number of parameters were extracted from each recorded airgun pulse: D duration, defined as the time between the -10 dB points relative to the peak of the envelope of the wave form [32,33]; L_pp_, received sound pressure level peak to peak; L_eq-fast_, root mean squared (RMS) averaged received sound pressure level over the 125 ms window with most sound energy [34]; SEL, sound exposure level, the pressure-squared time integral over a 1 second window around the pulse [3]; f_peak_: peak frequency of the power density spectrum; BW-_3dB_: frequency bandwidth defined as the separation between 3 dB points relative to the peak of the power density spectrum; BW-_10dB_: frequency bandwidth defined as the separation between 10 dB points relative to the peak of the power density spectrum.

#### Transmission loss

A geometric transmission loss model was estimated based on received levels on all six recording distances and individually for all recording sets (combinations of airgun volume and pressure). We used a least squares fit of the slope parameter к with the transmission loss, range r given as кlog(r), i.e. ignoring the frequency dependent absorption over the short ranges considered here. Absorption losses are less than 1 dB/km for frequencies below 10 kHz [25] and frequencies above 10 kHz only contribute negligibly to the overall energy of the pulses.

#### Audiogram weighting

Frequency weighting of signals is increasingly used to accommodate the fact that marine mammals have differently shaped audiograms [3,34,35]. This weighting should be species specific and address the fact that the hearing of an animal is not equally sensitive at all frequencies. At least two different approaches to weighting can be taken: based on audibility (audiogram weighting [34,36]) or based on perceived loudness [3,37,38]. We have selected to use audiogram-based weighting, as it is more consistent with experimental data than loudness weighting for harbor porpoises [34]. Accordingly, weighting functions were fitted to audiograms from porpoises [39,40] and a harbor seal [41] using the following expression:

$$\begin{array}{l} W_{f}=offset-20\;\text{log}\;w_{f}\\ w_{f}=\frac{f_{high}{}^{2}\cdot f^{2}}{\left(f^{2}+f_{high}{}^{2})\cdot (f^{2}+f_{low}{}^{2}\right)} \end{array}$$

where *offset*, *f*_*high*_ and *f*_*low*_ were set to 60 dB, 1 kHz and 100 kHz respectively for harbor seal and 45 dB, 10 kHz and 300 kHz respectively for harbor porpoise, according to a line of best fit of the audiograms (S1 Fig). Weighted sound exposure levels could then be found as:

$$SEL_{weighted}=10\;\text{log}(d\Delta f\sum_{0}^{Fs/2}{\left(p_{f}w_{f}\right)}^{2})$$

where p_f_ is the absolute power spectral density at frequency f, d is the duration of the pulse, Δf is the analysis bandwidth of the power spectrum and Fs is the sampling frequency of the recording.

## Results

**Fig 2:**
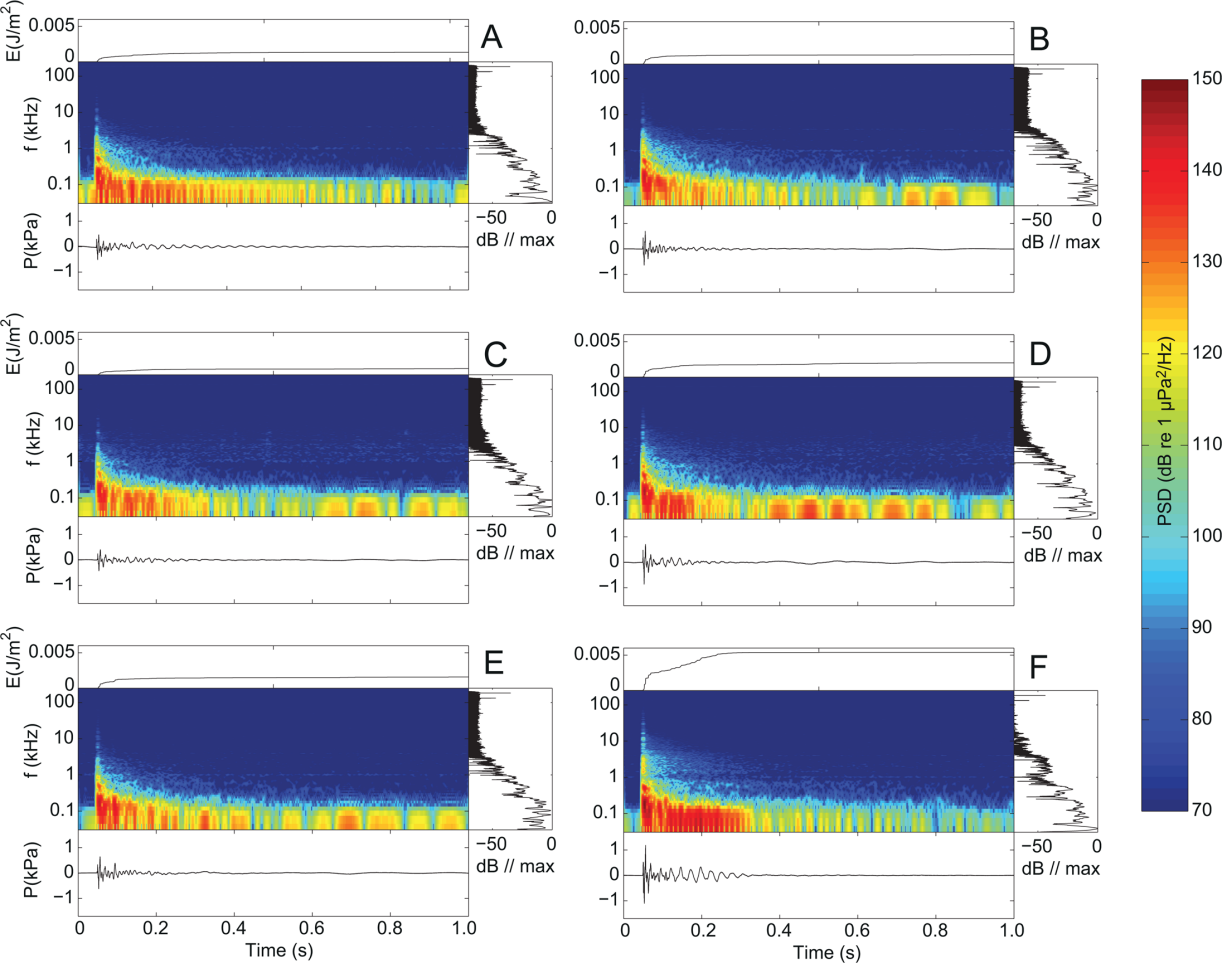
Effect of airgun volume and operating pressure on source characteristics. Shown are representative recordings obtained 120 m from the airgun at different combinations of volume (from top to bottom: 10 in^3^, 25 in^3^ and 40 in^3^) and operating pressure (left: low; right: high). Each plot contains four subplots with different representations of the signal. Bottom panel: time signal. Center panel: spectrogram (power spectral density, PSD) with logarithmic frequency axis (common color scale across subplots A-F at extreme right). Right panel: normalized power spectral density plotted on the same vertical frequency axis as the spectrogram. Top panel: cumulated sound energy of the pulse from start to end of recording.

The recorded airgun pulses contained most energy at low frequencies (Figs 1 and 2) with peak frequencies between 5 and 90 Hz (Table 1). However, considerable energy was also present at frequencies well beyond 10 kHz even at 1300 m, the largest recording range in this study (cf. Fig 1F). The recorded waveforms consisted of a short main pulse followed by a negative surface-reflected pulse (sometimes referred to as the ghost arrival [6]) and a subsequent long tail caused by sequential bubble formation and collapses, as well as refractions and reflections from the surface and sea floor (most evident in Fig 2F, bottom panel). The spectrograms (Fig 2, center panels) show interference patterns (repeating gaps in the spectrograms) caused by multipath propagation of the signal.

**Table 1:** Source characteristics of the airgun operated at different combinations of pressure and volume.

	10 in^3^ airgun		25 in^3^ airgun		40 in^3^ airgun	
	low	high	low	high	low	high
**D-duration (ms)**	19	20	40	21	48	21
	38	20	76	20	50	21
	84	18	78	53	49	21
	167	19	76	100	48	21
**L**_**pp**_ **(dB re 1μPa)**	180	182	178	184	182	187
	179	183	179	183	182	188
	178	183	178	184	182	188
	176	182	179	184	182	188
						188
**L**_**eq-fast**_ **(dB re 1μPa)**	161	161	159	163	162	167
	160	162	160	163	162	167
	160	161	160	164	162	167
	159	161	160	165	162	168
						168
**SEL (dB re 1μPa** ^**2**^ **s)**	152	152	151	154	153	159
	153	153	151	155	154	160
	153	152	151	155	153	159
	152	153	151	156	153	159
						159
**f**_**peak**_ **(Hz)**	34	113	89	6	6	39
	36	113	90	46	5	40
	38	5	90	42	79	40
	39	5	90	8	79	40
						40
**BW**_**-3dB**_ **(Hz)**	3	111	86	41	103	3
	3	7	56	5	0	4
	3	110	86	3	3	4
	3	3	57	84	3	4
						5
**BW**_**-10dB**_ **(Hz)**	64	211	172	152	185	47
	36	206	173	150	101	151
	39	240	146	178	154	152
	8	160	170	152	154	120
						154

Unweighted values are given for 4–5 recordings of airgun pulses for different combinations of airgun volume and output pressure obtained at a range of 120 m from the airgun.

**Fig 3:**
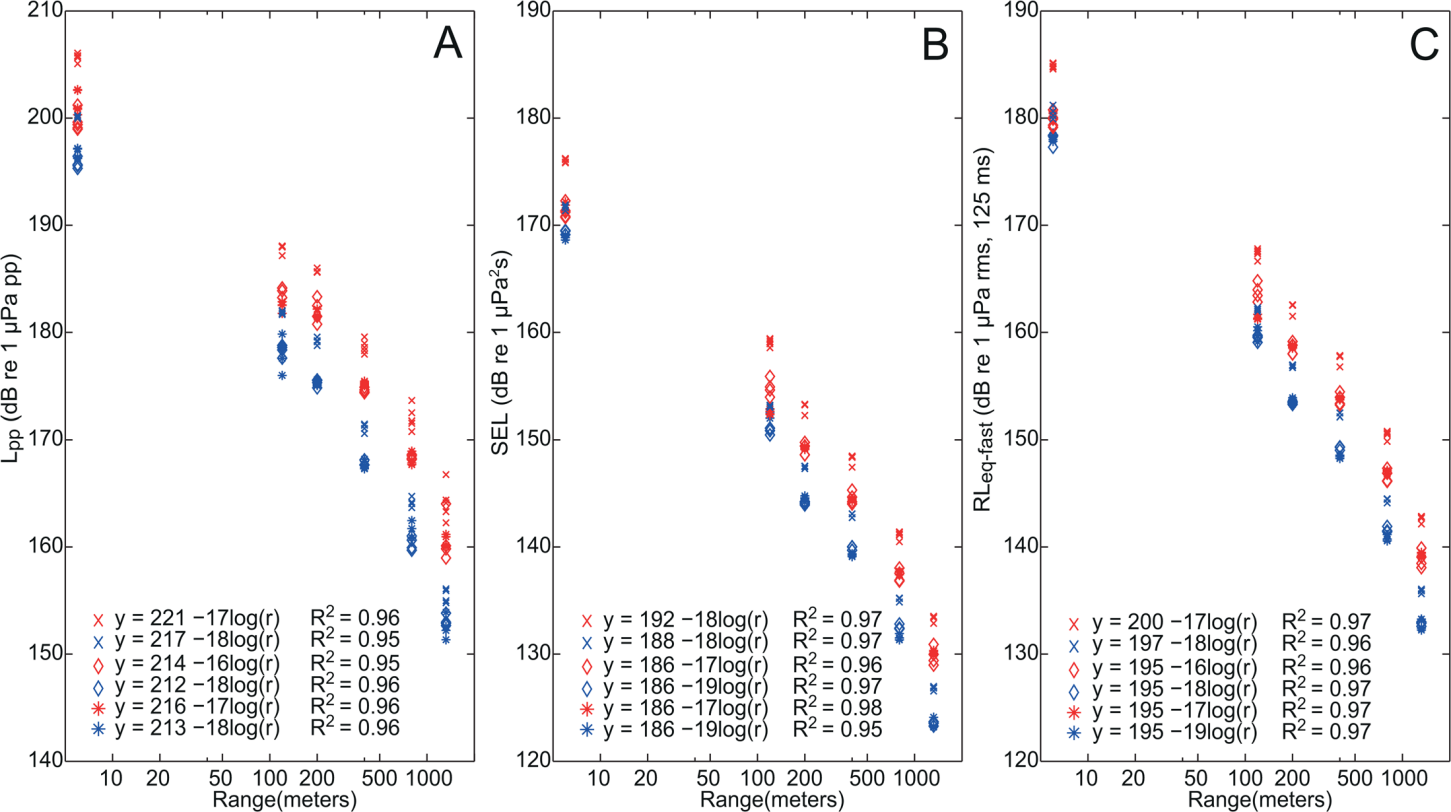
Transmission loss. Exposure levels estimated as L_pp_ (panel A), SEL (panel B) and L_eq-fast_ (panel C) as a function of distance to the airgun. Linear transmission loss models, кlog(r), estimated by linear regression of values from the six stations (6 m, 120 m, 200 m, 400 m, 800 m and 1300 m) are given for all six combinations of airgun volume (10 in^3^, 25 in^3^ and 40 in^3^) and pressure (high: red, and low: blue). The intercept with the y-axis approximates the back-calculated source levels (at 1 m), as shown next to the linear regression lines.

A comparison of the three airgun volumes at low and high pressure (Fig 2 and Fig 3 and Table 1) shows airgun characteristics changing with firing pressures and to a smaller degree with volume; sound levels of recordings at a range of 120 m (Table 1) show that L_pp_ range from 176 dB re 1 μPa to 188 dB re 1 μPa, whereas SEL range between 151 and 160 dB re 1 μPa^2^s and L_eq-fast_ range between 159 and 168 dB re 1 μPa, with higher values for larger volume and higher pressure.

Source levels at 1 m from the airgun were approximated in Fig 3 to 212–221 dB re 1 μPa (L_pp_), 186–192 dB re μPa^2^s (SEL) and 195–200 dB re 1 μPa (L_eq-fast_) again depending on size and volume of the airgun. The corresponding levels recorded at a range of 1300 m from the airgun were approx. 60 dB lower being 152–167 dB re 1 μPa (L_pp_), 123–133 dB re μPa^2^s (SEL) and 132–143 dB re 1 μPa (L_eq-fast_).

### Propagation of airgun pulses in shallow water

Unweighted L_pp_, SEL and L_eq-fast_ values are shown for the three airguns at low and high pressure at six ranges (station 1–6) in Fig 3. Linear regressions based on these recordings show transmission loss coefficients (к) between 16 and 19 dB per 10-fold increase in distance, which are below predictions from simple spherical spreading loss (к = 20), but considerably above predictions from cylindrical spreading loss (к = 10). Spherical and cylindrical spreading loss models are commonly used as rough estimations of sound propagation in deep and shallow waters, respectively [25].

**Fig 4:**
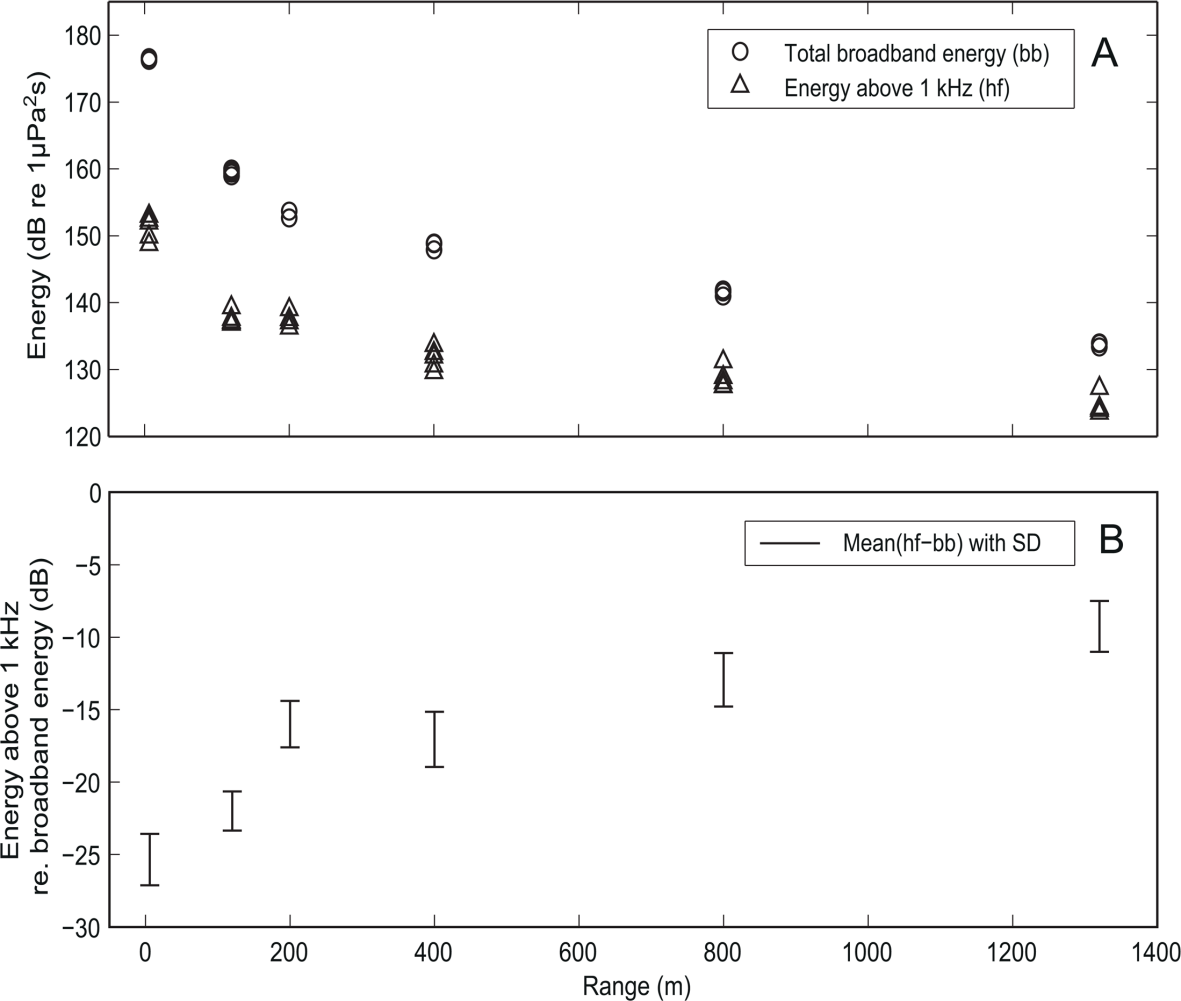
High-pass filtering with transmission range. A. Total broadband energy in all frequency bands (circles) and summed energy above 1 kHz (triangles) as a function of range shown for all recordings of the 40 in^3^ airgun at high pressure. B. Relative values (expressed in dBs) of energy above 1 kHz compared to the total broadband energy shown as means with standard deviations (SD).

At the two closest recording ranges (Fig 1A and 1B), all frequency components in the broadband main pulse of the airgun signal arrived simultaneously, whereas higher frequencies arrived earlier than the low frequencies at larger ranges (Fig 1C–1F) creating a slight frequency sweep in the main pulse. Furthermore, the peak frequency increased with range (Fig 1A–1F), as did the noise energy above 1 kHz relative to the total broadband energy (Fig 4). At close ranges the energy above 1 kHz amounted to less than 1% of the total energy (23–27 dB below broadband energy), whereas it constituted up to 20% (7–11 dB below broadband energy) of the total at a range of 1300 m from the airgun due to the high pass filter effect of shallow water (Fig 4).

### Audiogram-weighted sound exposure level

**Fig 5:**
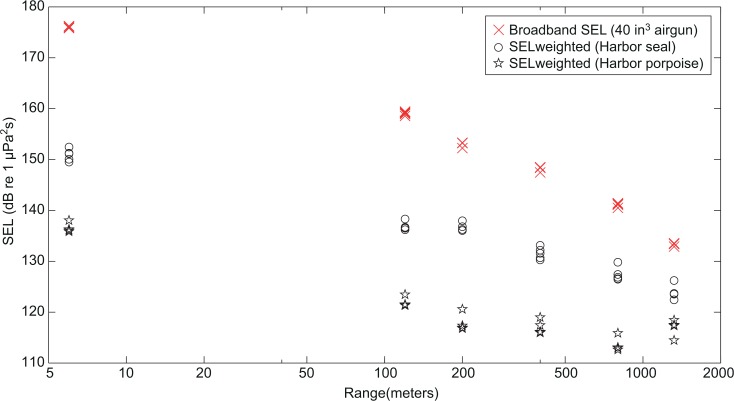
Audiogram-weighted sound exposure levels (SELs). Unweighted SELs are shown for all recording ranges for broadband pulses from a 40 in^3^ airgun volume at high output pressure (crosses), together with the audiogram-weighted SELs for harbor seal (circles) and harbor porpoise (stars).

Audiogram-weighted sound exposure levels were higher for seals (122–152 dB re μPa^2^s) than for porpoises (113–135 dB re μPa^2^s), reflecting the generally better hearing of seals at low frequencies (Fig 5). At short ranges the differences between unweighted and weighted values were large (24–38 dB) in comparison to what was found at longer ranges, where weighted levels were closer to unweighted values (differences between 11 and 19 dB). This is due to the high pass filtering of the signals with increased transmission range, as the low frequencies, where seals and porpoises have poor hearing, were filtered out by the shallow water transmission channel.

## Discussion

### Characteristics and propagation of airgun pulses

Here we show that a single airgun can generate high level broadband pulses propagating horizontally away from the airgun in shallow water (Figs 1 and 3). As expected, exposure levels increase with firing pressure and airgun volume with levels emitted by the 40 in^3^ airgun being approximately 6 dB higher than for the 10 in^3^ airgun at high output pressure (Table 1). This is consistent with output pressure being proportional to the cube root of airgun volume [6]. However, the 25 in^3^ airgun did not follow this relationship, as there was only a small difference in exposure levels from the 10 in^3^ and the 25 in^3^ airgun (Fig 3). This deviation may be due to a difference between the actual firing airgun volumes and the nominal volumes by the manufacturer and/or the fact that the highest output pressure for the 25 in^3^ airgun (114–117 bar) was slightly lower than for the two other volumes (120–122 bar). Peak to peak source levels back-calculated to 1 m were 212–221 dB re 1 μPa (pp), comparable with the source level of 222 dB re 1 μPa (peak) for a 40 in^3^ airgun reported by Greene and Richardson [28], but considerably lower than source levels of large airgun arrays of up to 259 dB re 1 μPa (pp) [4]. Despite the complex properties of shallow water habitats, our recordings demonstrate that in this area broadband airgun pulses propagated with a transmission loss slightly lower than predicted from simple spherical spreading over moderate ranges (out to 1300 m) approximately following 18log(r) (Fig 3). Environmental properties of other shallow water habitats with similar water depths may cause propagation patterns that deviate from this [25]. However, when examining the propagation of noise in different frequency bands, it is evident that the shallow water does have a considerable effect on the spectral composition of the airgun pulse.

For all three airgun volumes at low and high output pressures, most of the signal energy was found at frequencies below 1 kHz (Figs 2 and 4). Yet, high frequency components were clearly present in the signals, even up to 10 kHz at the longest recording range (1300 m)(Fig 1F), in line with the findings by Goold and Fish [14]. Results also show that the ratio between high frequency and low frequency noise changed with distance, so that the proportion of signal energy above 1 kHz relative to the total signal energy increased with distance to the airgun source (Fig 4). This can be explained by the generally poor propagation of low frequencies in shallow water transmission channels due to interference with both the sea surface and the seafloor [25]. Shallow waters therefore effectively function as high pass filters on the signal with a cut-off frequency that depends on the water depth and the sediment type. A related phenomenon, time stretching [25], is also illustrated by our recordings. By time stretching, where lower frequency parts of the signal propagate slower than high frequencies, a downward frequency sweep was created in the main pulse (Fig 2E and 2F), as also seen in Greene and Richardson [28]. These findings highlight that while overall sound pressure levels and sound exposure levels could be reasonably well predicted by simple transmission loss models for the short ranges considered here, the actual propagation of airgun noise in shallow water was complex. Good predictions of the spectral and temporal properties can therefore not be made without a proper sound propagation model that can take the full complexity of the sound transmission channel into account for longer ranges than considered here.

### Effects on marine mammals

Airguns are used extensively worldwide for seismic surveys, either individually or in small arrays for surveys probing only the top layers of the sediment (sub-bottom profiling), or in large arrays for surveys probing many kilometers into the ocean crust [1,7]. As the use of seismic equipment overlaps with many important habitats for marine mammals worldwide [31], many marine mammals are regularly exposed to airgun noise and there is a large and recognized potential for adverse effects (e.g. [1,23,42,43]). The present results confirm that there are substantial medium-to-high frequency components in airgun pulses, overlapping with the range of best hearing of harbor seals and harbor porpoises [39,40,41]. These medium-to-high frequency components are not just present at close range, but out to at least 1300 m (the largest range used in this study). This indicates that both harbor porpoises and harbor seals may be affected by even a single airgun. In addition, our results highlight the importance of examining spectral characteristics of propagating anthropogenic noise sources when assessing the impact on marine mammals, as the potential effects are highly dependent on the frequency content of the noise. Potential effects on marine mammals exposed to seismic noise may include behavioral disruptions, acoustic masking and temporary or permanent threshold shift (TTS or PTS). However, we still know very little about how marine mammals respond to airgun pulses and how a variety of other factors (e.g. season, habitat and previous exposure) influence these responses [31,43]. This calls for additional research to elucidate how a wide range of toothed whales and pinnipeds are affected by seismic noise to inform relevant mitigation measures. The following discussion should be read with that current lack of detailed information in mind.

By comparing the recorded characteristics and propagation of airgun pulses in this study with previous studies on the hearing and response thresholds of harbor porpoises and harbor seals to anthropogenic underwater noise, we can get a crude estimate on how these animals might be affected by pulses from a single airgun in a shallow water habitat. This approach is conservative as many airguns used in shallow waters are likely to be larger in size than the airguns used in this study and since most airguns will be used in arrays of multiple airguns. For airguns used in deeper waters, the ratio of high frequency energy to broadband energy may be smaller than shown here, as there is better propagation of lower frequency sounds in deep water in comparison to in shallow waters (but for example of a surface duct see [15,17]). However, the high frequency energy emitted from the omnidirectional airgun(s) will be unaffected by the change in depth.

When it comes to auditory impairment, a small temporary threshold shift (TTS) can be used as a precautionary proxy for onset of impact, which is useful because several studies on TTS now exist. Although no studies are available where TTS was induced in seals by sounds similar to airgun pulses, a few studies have looked at TTS from exposure to low frequency octave band noise. Kastak et al. [44] investigated long duration exposure of three species of pinnipeds to octave-band noise centered at 2.5 kHz and determined a threshold for eliciting 6 dB of TTS at a cumulated SEL of 182 dB re 1μPa^2^s. Kastelein et al. [45] found slightly lower thresholds (cumulated SEL between 170 and 179 dB re 1μPa^2^s) when exposing harbor seals to noise centered on 4 kHz. If complying with the equal energy hypothesis [3,46], this would mean that only a seal within a few meters of the 40 in^3^ airgun recorded in this study would risk suffering TTS from a single exposure (cf. Fig 3B). However, airgun pulses are usually emitted in succession resulting in cumulated exposures, which could potentially lead to TTS at lower exposure levels of individual pulses, as the energy of pulses is cumulated. Therefore TTS could be expected at longer ranges than for a single pulse, as a result of cumulative exposure. Assuming a complete summation of energy, the SELcum for 100 pulses is 20 dB higher than SELcum for a single pulse [3]. If then assuming that a seal remains at the same range from the airgun, this would mean that the maximum range where TTS may occur increases roughly with a factor of 10 to some tens of meters, as the SELcum at this range is predicted to be 18 dB (18log10 from Fig 3) lower. In any case, the risk of inflicting TTS on harbor seals by the use of single airguns seems very low, unless a seal remains very close to the airgun for a long time. The situation is somewhat different for harbor porpoises, where a study has shown that 6 dB of TTS could be induced in a porpoise by exposing it to a single airgun pulse with an SEL of 164 dB re μPa^2^s [47]. For the single airgun considered here, this onset threshold corresponds to a porpoise experiencing a 6 dB TTS at 35 m from the airgun (interpolating the data from Fig 3B). This distance will increase if more than one pulse is used, as is usually the case, but recent data indicates that the threshold for summed energy of multiple pulses in harbor porpoises is higher than for single pulses, contrasting with the equal energy hypothesis [48]. Therefore a repetition of the calculation for the harbor seal, which would indicate a maximum range for TTS of roughly 350 m for 100 pulses in a porpoise, is likely overestimating the true range for inflicting TTS in this species. Consequently, the ranges where TTS can be inflicted with a small single airgun are short, on the order of some hundred meters, under the unrealistic assumption that the porpoise does not move in response to the sound. In reality, a porpoise is likely to begin moving away from the sound source upon hearing the first pulse, thereby effectively reducing the cumulated exposure.

Besides auditory impairment, behavioral responses (e.g. avoidance, changes in feeding behavior or altered dive times) may also have serious effects on the fitness of an animal. For example, a short exposure to a sound may cause a lowered foraging efficiency, a poorer ability to navigate or a lowered communication with conspecifics, and in a worst case scenario momentarily inattentiveness can result in a failure to detect a predator or a gill net, with fatal consequences for the individual [49]. Few studies have quantified the response thresholds of seals to impulsive sounds. Thompson et al. [19] studied harbor seals exposed to airgun pulses (source levels of 215–224 dB re 1 μPa pp) using a system very similar to the one of the present study, and found a strong avoidance response within 20–100 m, with animals exhibiting bradycardia and ceasing to feed. Blackwell et al. [50] studied the reactions of ringed seals to pile driving noise (comparable to airgun noise in being powerful, impulsive and with low frequency emphasis) and found no detectable reactions at estimated received peak levels as high as 157 dB re 1 μPa and single pulse SELs of 145 dB re 1 μPa^2^s, indicating that reactions in case of a 40 in^3^ airgun would not be expected unless seals of this species are closer than 1 km (cf. Fig 3).

Although seals have better hearing than odontocetes at low frequencies, where most of the energy in airgun noise is present, previous response studies have indicated that odontocetes are generally more sensitive to seismic noise than pinnipeds [23,31]. Based on avoidance reactions of harbor porpoises to pile-driving noise reaction thresholds for porpoises have been estimated at received levels between 141 and 149 dB re 1 μPa (Leq-fast, unweighted [34]). These findings, in conjunction with the broadband characteristics and high noise levels of airguns recorded in the present study, indicate that porpoises could react by negative phonotaxis several kilometers from even a single 40 in^3^ airgun (cf. Fig 3). Thompson et al. [51] studied effects of seismic noise on wild harbor porpoises and found no long term displacement response. Yet, the authors highlight that smaller scale effects, such as a change in foraging performance should be investigated further as such effects may have serious fitness consequences for an animal. The authors also highlighted that displacement effects may depend highly on habitat quality. This is consistent with the study by Gordon et al. [52] that found porpoises staying in a preferred habitat during exposure to a small airgun source. Harbor seals have also been observed tolerating strong noise pulses if the area is attractive for feeding or reproduction [53]. Pirotta et al. [20] suggest that responses to seismic noise is a trade-off between perceived risk and cost of leaving a favorable habitat, as they found that porpoises remaining in the area reduced buzzing activity by approx. 15% in the presence of seismic noise reflecting decreased foraging activity with potential serious impact on their energy budgets.

### Implications for mitigation

In most countries, current mitigation measures to reduce the risk of injury from airguns include a shut-down zone of some hundred meters around the survey ship; 500 m in case of the UK regulation [42]. If marine mammals are sighted within this zone the seismic activity must be stopped. Our findings here suggest that harbor porpoises and harbor seals are unlikely to experience TTS at distances beyond 500 m from a single airgun, and thus a 500 m shut-down zone should be sufficient to avoid auditory injury in these two species for small airguns. TTS effects of larger airgun arrays would be more severe given the higher output levels, but an estimate of the impact ranges cannot be assessed here. Such estimates would require thorough measurements in the specific habitats where such arrays are used, as the propagation and characteristics of airgun pulses are highly dependent on environmental properties and the array configuration of the airguns. The present study also shows that even at 1300 m from a single airgun, noise levels are elevated over a broad frequency range, up to at least 10 kHz with audiogram-weighted levels above 113 dB for harbor porpoises and 122 dB for harbor seals. Thus, animals beyond this often used shut-down zone of 500 m will suffer from unmitigated, but likely substantial behavioral disruptions even for the small airguns considered here.

Anthropogenic noise is quantified by a range of different measures, some of which are more relevant than others, when assessing the impact on marine mammals. For seismic noise, we advocate that noise levels should be quantified as RMS sound pressure levels over a specified short time window matching the integration time of the mammalian ear, such as L_eq-fast_ (125 ms window [34]), by the sound exposure level (SEL) or at least by the duration independent peak-peak measure [54]. Furthermore, as an alternative to fixed mitigation distances, exposure measures to approximate TTS and PTS onsets have been developed for different species groups in the recent acoustic guidance report from the National Oceanographic and Atmospheric Administration (NOAA)[35]. This is an important step towards mitigating auditory injuries in marine mammals. However, as these criteria are based on data from only a few species, it is not yet known how well the exposure criteria reflect actual thresholds for a wide range of species. To better assess how anthropogenic noise influences an animal, noise should be weighted according to the hearing sensitivity of relevant species, as also highlighted by NOAA [35]. In this study, audiogram-weighted levels were considered to give a better understanding of the noise as perceived by animals, in line with the A-weighted sound pressure levels ubiquitous in human community noise regulation. Audiogram-weighted sound exposure levels here were found to be between 122–152 dB re μPa^2^s for harbor seals and 113–135 dB re μPa^2^s for harbor porpoises. At all ranges (6–1300 m) audiogram-weighted levels were shown to be higher for seals, as a consequence of their better low frequency hearing compared to porpoises, where most of the airgun energy is. Audiogram-weighted noise levels are promising measures for impact assessments, but require audiograms for various marine mammal species along with good characterizations of the anthropogenic noise sources. Furthermore, although environmental regulations in general acknowledge that disturbances of animals should be avoided (e.g. [55,56]), the complex behavioral responses of marine mammals to noise exposure complicates formation of impact assessments. Such assessments require detailed knowledge about how different species respond to noise under changing conditions, including different seasons and depending on the activity and previous exposure of the animal [1,49]. As a rough estimate for harbor porpoises, Tougaard et al. [34] suggest to approximate impact on porpoises by an exposure limit 45 dB above the hearing threshold for negative phonotaxis, and an exposure limit for onset of TTS expressed as SELcum and equal to the hearing threshold plus 100–110 dB. This calculation may seem inappropriate, as SELcum is expressed in units of energy (μPa^2^s) whereas the hearing threshold is expressed in units of pressure (μPa), but he comparison is valid, however, if one considers SEL as a normalization to a 1 second signal, in which case the rms-averaged sound pressure equals SEL.

Regardless of the method for assessing impact, mitigation measures should follow the precautionary principle to account for the cumulative impact of multiple anthropogenic stressors on a population, such as intense shipping and construction of wind farms, which together with the use of airguns are prevalent sources of noise in shallow coastal waters inhabited by many species of marine mammals.

## Supporting Information

S1 Fig Weighting functions fitted to audiograms. Underwater hearing thresholds of a harbor seal (Reichmuth et al. 2013, plot A) and a harbor porpoise (Kastelein et al. 2010, plot B) shown with the fitted curves used to estimate audiogram-weighted sound exposure levels in Fig 5.
(PDF)
