# Supplementary figures and images for: Characteristics and Propagation of Airgun Pulses in Shallow Water with Implications for Effects on Small Marine Mammals
Source: PLoS One. 2015 Jul 27;10(7):e0133436. doi: 10.1371/journal.pone.0133436 (PMC4516352; doi:10.1371/journal.pone.0133436)

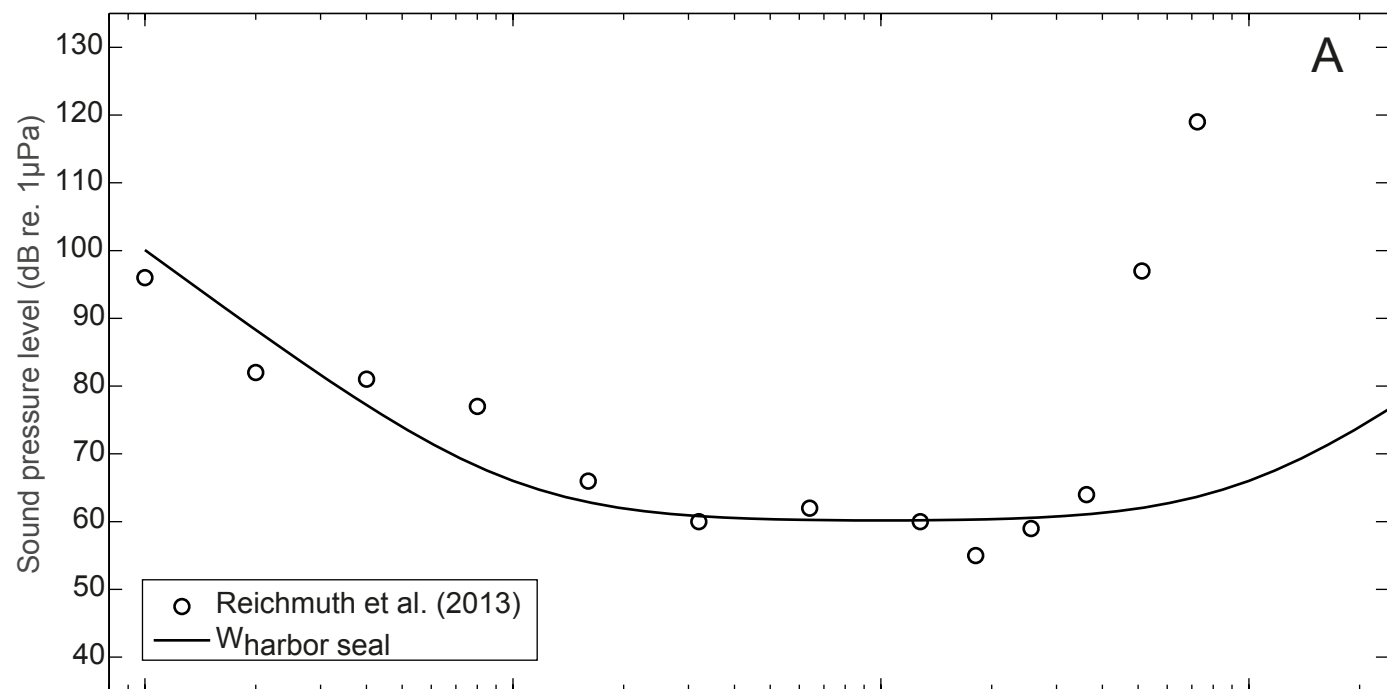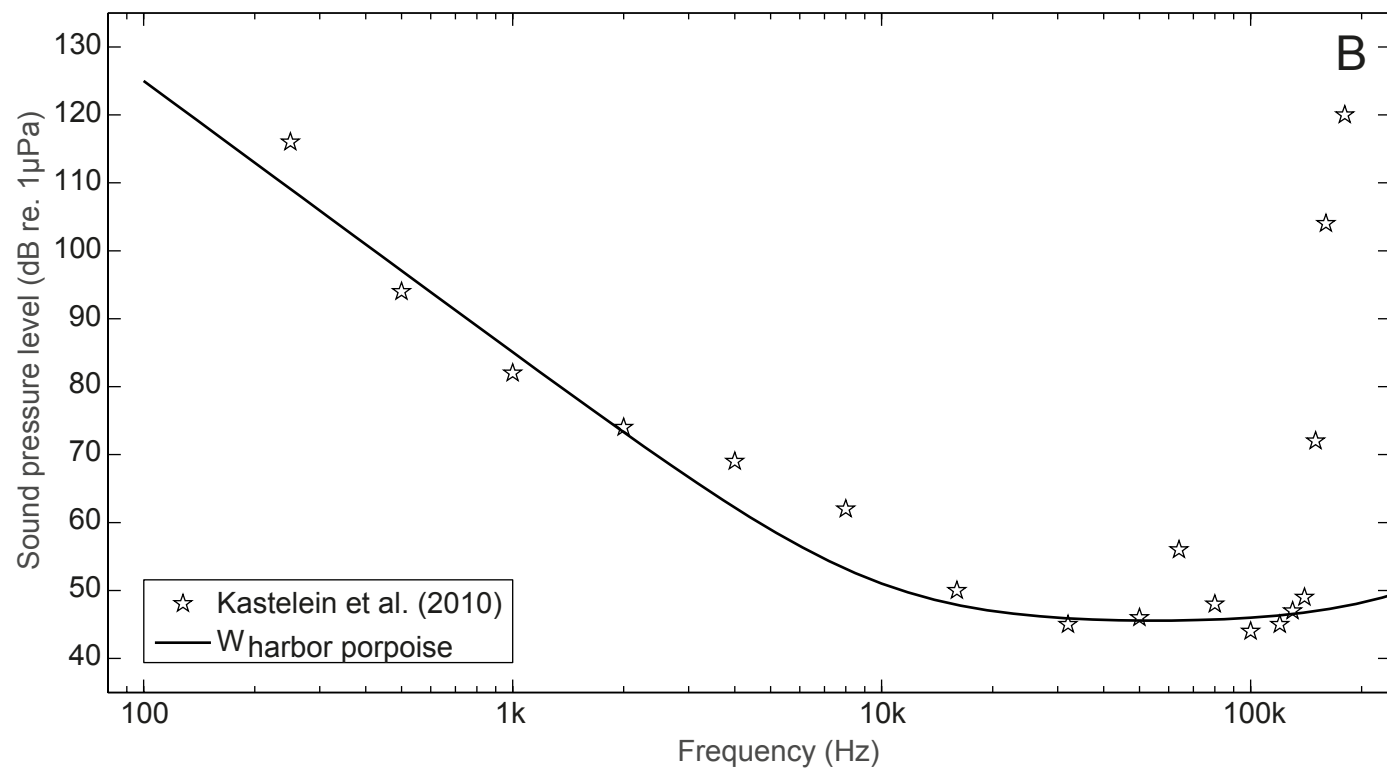

Supplement: S1 Fig — Underwater hearing thresholds of a harbor seal (Reichmuth et al. 2013, plot A) and a harbor porpoise (Kastelein et al. 2010, plot B) shown with the fitted curves used to estimate audiogram-weighted sound exposure levels in Fig 5. (PDF) [file pone.0133436.s001.pdf]
